# The neural and molecular basis of working memory function in psychosis: a multimodal PET-fMRI study

**DOI:** 10.1038/s41380-019-0619-6

**Published:** 2019-12-04

**Authors:** Faith Borgan, Owen O’Daly, Mattia Veronese, Tiago Reis Marques, Heikki Laurikainen, Jarmo Hietala, Oliver Howes

**Affiliations:** 1 Psychosis Studies Department, Institute of Psychiatry, Psychology and Neuroscience, King’s College London, London, England; 2 Institute of Clinical Sciences, Faculty of Medicine, Imperial College London, Hammersmith Hospital, London, England; 3 Centre for Neuroimaging Sciences, Institute of Psychiatry, Psychology and Neuroscience, King’s College London, London, England; 4 Turku PET Centre, Turku University Hospital, Turku, Finland; 5 Department of Psychiatry, University of Turku and Turku University Hospital, Turku, Finland

**Keywords:** Molecular biology, Neuroscience, Biological techniques, Schizophrenia, Prognostic markers

## Abstract

Working memory (WM) deficits predict clinical and functional outcomes in schizophrenia but are poorly understood and unaddressed by existing treatments. WM encoding and WM retrieval have not been investigated in schizophrenia without the confounds of illness chronicity or the use of antipsychotics and illicit substances. Moreover, it is unclear if WM deficits may be linked to cannabinoid 1 receptor dysfunction in schizophrenia. Sixty-six volunteers (35 controls, 31 drug-free patients with diagnoses of schizophrenia or schizoaffective disorder) completed the Sternberg Item-Recognition paradigm during an fMRI scan. Neural activation during WM encoding and WM retrieval was indexed using the blood-oxygen-level-dependent hemodynamic response. A subset of volunteers (20 controls, 20 drug-free patients) underwent a dynamic PET scan to measure [^11^C] MePPEP distribution volume (ml/cm^3^) to index CB1R availability. In a whole-brain analysis, there was a significant main effect of group on task-related BOLD responses in the superior parietal lobule during WM encoding, and the bilateral hippocampus during WM retrieval. Region of interest analyses in volunteers who had PET/fMRI indicated that there was a significant main effect of group on task-related BOLD responses in the right hippocampus, left DLPFC, left ACC during encoding; and in the bilateral hippocampus, striatum, ACC and right DLPFC during retrieval. Striatal CB1R availability was positively associated with mean striatal activation during WM retrieval in male patients (*R* = 0.5, *p* = 0.02) but not male controls (*R* = −0.20, *p* = 0.53), and this was significantly different between groups, *Z* = −2.20, *p* = 0.02. Striatal CB1R may contribute to the pathophysiology of WM deficits in male patients and have implications for drug development in schizophrenia.

## Introduction

Verbal working memory (WM) deficits are a stable component of the cognitive deficit seen in schizophrenia [1] that predict poor clinical [2] and functional outcomes [3]. However, the neurobiology underlying WM impairments remains poorly understood and unaddressed by current treatments [4]. Identifying the pathophysiology underlying impairments in WM is therefore important for the development of pharmacological treatments targeting WM deficits in schizophrenia.

WM, the temporary storage and manipulation of information, is comprised of distinct processes including encoding, maintenance and retrieval [5]. Studies using the Sternberg Item-Recognition paradigm (SIRP), able to disentangle the effects of WM encoding and WM retrieval, have shown that healthy volunteers show greater task-dependent blood-oxygen-level-dependent (BOLD) responses during WM encoding in the bilateral anterior cingulate cortex (ACC), ventral striatum, left hippocampus, right dorsolateral prefrontal cortex (DLPFC) and bilateral parietal cortex; and greater BOLD signal during WM retrieval in the bilateral parietal cortex, bilateral ttemporal cortex and the posterior cingulate [6].

While numerous studies have investigated WM in schizophrenia [7], few studies have used paradigms that are able to investigate WM encoding and retrieval mechanisms separately [8–11], and those that have, typically fail to model them separately [8–10] or do not report findings for both WM encoding and retrieval [11]. While one study investigated WM encoding and retrieval in anti-psychotic-treated, male patients with chronic schizophrenia [12], groups were not matched on performance.

In this context, anti-psychotic treated patients relative to controls showed lower activation during WM encoding in the inferior and middle frontal gyri, but greater activation during WM retrieval in the left hippocampus, left striatum and the right inferior and frontal gyri [12]. However, it is unclear if group differences [12] may be related to the effects of anti-psychotic medication, substance use, illness chronicity or poor task engagement.

Mice deficient in cannabinoid 1 receptors (CB1Rs) on GABAergic interneurons exhibit WM deficits [13]. Moreover, alterations in peripheral CB1R mRNA levels have been associated with poor cognitive performance [14]. We recently showed that patients with schizophrenia show fewer cortical CB1Rs, where lower levels are associated with poorer cognitive functioning [15]. Moreover, medication-naïve FEP patients, who do not use cannabis, show greater levels of the endogenous CB1R agonist, anandamide [16], shown to impair memory retrieval in rodents [17–21].

We aimed to investigate the neural basis of both WM encoding and WM retrieval, and their relationship to CB1R availability. We predicted that patients relative to controls would show lower CB1R availability [15, 22] and altered functional activation during WM encoding and WM retrieval [12]. Taking together findings that CB1R modulate synaptic transmission and plasticity underlying memory [23–25] and literature showing an association between CB1R availability and behavioural measures of cognition in schizophrenia [15], we predicted that CB1R availability would be associated with the neural correlates of WM.

## Methods

### Design

A cross-sectional design was used. The neural correlates of WM were investigated in males and females, as well as males alone. Due to sex differences in CB1Rs [26], we investigated CB1Rs in males, with the view of investigating females in future. The PET data, but not the fMRI or PET-fMRI relationships, were recently reported [15].

### Participants

Sixty-six volunteers including 31 patients with drug-naïve/free first episode psychosis (FEP) (mean [SD], age, 26.64 [4.68] years; 26 males, 5 females) and 35 healthy volunteers (mean [SD] age, 27.12, [5.32] years; 26 males, 9 females) matched on age (age ±3 years) and sex were included. Patients were recruited from early intervention services for psychosis and healthy volunteers were recruited via local advertising in London, United Kingdom. A power calculation indicated that a sample size of 20 volunteers per group would have >80% power to detect a relationship of *R*^2^ = 0.35, *p* < 0.05 (two-tailed) (see supplementary for full details).

### Inclusion and exclusion criteria

Patients met the following criteria: (1) <3 years of illness onset; (2) mental capacity to consent; and (3) diagnosis of schizophrenia/schizoaffective disorder [27]. Healthy volunteers met the following criteria: (1) no current/lifetime history of an Axis I disorder, as determined by the Structured Clinical Interview of DSM-IV-TR Axis I Disorders (SCID) [27]; (2) and no family history (first/second-degree) of an Axis I disorder [28].

Exclusion criteria for all volunteers were as follows: (1) current/lifetime history of substance abuse/dependence, as determined by the SCID [27]; (2) substance use within the last month; (3) positive result on a urine toxicology test detecting THC metabolites for up to 30 days (50 ng/ml cut off) or a positive result on a test detecting cocaine, amphetamine, cannabis, opiates and benzodiazepines; (4) head injury leading to loss of consciousness; and (5) contraindications to MRI safety.

### Measures

#### Clinical and demographic variables

Age, sex, ethnicity, current/previous alcohol, nicotine and illicit substance use, age of illness onset/duration were recorded. Clinical symptom severity was determined using the Positive and Negative Syndrome scale (PANSS) [29].

#### Neuroimaging

##### Sternberg Item-Recognition Paradigm (SIRP)

High-resolution T1-weighted images and the SIRP were acquired on a General Electric MR750 3.0 tesla scanner (see supplementary materials for neuroimaging acquisition parameters). The SIRP, shown to have good reliability [30], was used to investigate WM [31]. The task comprised of (1) encoding trials, where volunteers were instructed to memorize sets of letters; (2) retrieval trials, where volunteers indicated whether they had seen the letters previously; and (3) rest trials (see supplementary materials for details and Supplementary Fig. 1 for a paradigm schematic).

##### Cannabinoid 1 receptor availability

As reported elsewhere [15], a CB1-selective radiotracer, [^11^C]MePPEP using arterial blood sampling, was used to measure CB1R availability [32] (see supplementary materials for neuroimaging acquisition parameters).

### Statistical analyses

Statistical analyses were conducted using Statistical Package for the Social Sciences (SPSS; Version 22) [33]. Data normality was assessed using the Shapiro−Wilk test; and equality of variances were assessed using the Lavene’s test.

#### Behavioural data analysis

Group differences in categorical and continuous variables were determined using chi-square and independent samples *t* tests, respectively.

#### fMRI analysis

Data were analysed using Statistical Parametric Mapping software (SPM-12; Version 6684) [34] using Matlab 8.5 [35]. Frame-wise displacement was calculated used methods described previously [36]. High velocity motion spikes were regressed out by including scan nulling (censoring) regressors for volumes with volume-to-volume frame-wise displacement greater than 0.5 mm. A standard pre-processing pipeline was implemented (see supplementary materials for methods). The blood-oxygen-level-dependent (BOLD) response was modelled using an event-related design where a canonical hemodynamic response function (HRF) was convolved with regressors encoding the onset and duration for the following ten conditions: encoding load (EL) 1, EL 3, EL 5, EL 7, EL 9, retrieval load (RL) 1, RL 3, RL 5, RL 7, and RL 9. Rest trials were left un-modelled and served as an implicit baseline. Individual fixed-effects analyses were performed for each participant to identify regional differences in relative activation using the following linear contrasts of parameter estimates: EL 3-1, 5-1, 7-1 and 9-1 and RL 3-1, 5-1, 7-1 and 9-1. To investigate group differences in the neural correlates of WM, a 2 (group: patient vs. control) × 4 (load: 3-1, 5-1, 7-1 and 9-1) ANOVA was conducted for encoding and retrieval, respectively, controlling for age, sex and mean frame-wise displacement. Independent samples *t* tests were also used to investigate group differences in (1) mean frame-wise displacement and (2) task response accuracy (% of overall correct responses).

Whole-brain analyses were conducted using the full sample (*N* = 66) including males and females, as well as males only (*N* = 52). Whole-brain and region of interest (ROI) analyses were repeated in male volunteers (*N* = 40) who had PET/fMRI, in order to permit the investigation of the association between CB1R and the neural correlates of WM in the same volunteers. ROI analyses were conducted for the ACC, hippocampus and striatum, defined using a standard probabilistic atlas [37]. Since this atlas [37] does not include the DLPFC, ROI analyses conducted for the DLPFC were defined using Brodmann areas 9 and 46 [38] using the WFU PickAtlas Toolbox (http://fmri.wfubmc.edu/software/pickatlas). These ROIs were selected based on findings that WM encoding activates the bilateral ACC, striatum, hippocampus and DLPFC in controls [6] and findings indicating that CB1R agonists administered to the striatum [39, 40], hippocampus [41, 42] and medial prefrontal cortex impair memory in rodents [17].

A result was deemed significant if it survived family-wise error (FWE) correction on the basis of the peak-level extent (pFWE < 0.05). Mean BOLD signal was extracted using the MarsBar toolbox (http://marsbar.sourceforge.net) using independently derived ROIs [37]. Mean BOLD signal for encoding and retrieval trials were extracted for whole-brain grey matter, defined using the WFU PickAtlas Toolbox (http://fmri.wfubmc.edu/software/pickatlas) in order to investigate data normality and equality of variances.

#### PET analysis

A standard pre-processing pipeline was implemented (see supplementary materials for methods). CB1R availability was indexed using the distribution volume (*V*_T_) of [^11^C]MePPEP using the Logan graphical method with a metabolite-free arterial plasma input function [43]. CB1R availability was investigated in the same ROIs that were used for the fMRI ROI analyses. A 2 (group) × 4 (region: DLPFC, ACC, hippocampus, striatum) repeated measures ANOVA was conducted to investigate group differences in CB1R availability.

#### PET and fMRI analyses

Pearson’s correlation coefficients were calculated to determine the association between CB1R availability and (1) performance accuracy; and (2) the linearity of the load-dependence of the BOLD response during WM encoding and WM retrieval (for each subject, we fitted a linear regression model (intercept and slope) and took the slope as our measure of linearity of the BOLD response, as a function of task difficulty (separately for encoding and retrieval)). Bonferroni corrections were applied. Levels of statistical significance were *p* < 0.05 for all tests (two-tailed).

## Results

### Demographic and clinical data

All data were normally distributed. There were no group differences in age, sex, ethnicity, socio-economic status, years of education, body mass index, current cannabis, alcohol or tobacco use (see Table 1).

**Table 1:** Sample clinical and demographic characteristics showing that there were no group differences between healthy volunteers and first episode psychosis patients in age, sex, ethnicity, socio-economic status, body mass index, cannabis, alcohol or tobacco use.

	Healthy volunteers	First episode psychosis patients	*t*/*x*^2^/*U*	df	*p*
*N*	35	31			
Age mean (SD), years	27.12 (5.32)	26.64 (4.68)	*t* = 0.52	64	0.60
Sex (male/female)	26/9	26/5	*x*^2^ = 1.18	2	0.55
Ethnicity (Caucasian/Black African or Black Caribbean/ Asian/ Mixed/missing)	15/3/11/4/2	12/0/6/3/0	*x*^2^ = 8.49	6	0.20
Years of education after compulsory education mean (SD)^a^	4.01 (3.81)	3.01 (2.59)	*t* = 1.63	58	0.11
Socio-economic status (high/medium/ low/unemployed/ student/missing data)^b^	3/4/10/1/13/4	1/2/13/5/7/3	*x*^2^ = 24.38	20	0.23
Body mass index mean (SD) score	25.12 (3.81)	25.65 (5.10)	*t* = −0.41	46	0.68
Current cannabis use (yes/no)	0/35	0/31	NA	NA	NA
Current alcohol use (yes/no/missing data)	21/12/2	17/13/1	*x*^2^ = 2.88	4	0.60
Current tobacco use (yes/no/ missing data)	10/23/2	13/17/1	*x*^2^ = 3.14	4	0.53
Diagnosis (schizophrenia/ schizoaffective disorder)	NA	28/3	NA	NA	NA
Illness duration mean (SD), months	NA	22.39 (12.80)	NA	NA	NA
Duration of prior treatment mean (SD), months	NA	6.16 (10.10)	NA	NA	NA
Current use of antipsychotics (yes/no)	NA	31/0	NA	NA	NA
Prior use of antipsychotics (yes/no)	NA	23/8	NA	NA	NA
PANSS positive mean (SD) score	NA	25.83 (14.90)	NA	NA	NA
PANSS negative mean (SD) score	NA	24.10 (7.53)	NA	NA	NA
PANSS general mean (SD) score	NA	40.45 (10.20)	NA	NA	NA
PANSS total mean (SD) score	NA	86.82 (21.83)	NA	NA	NA

*N* sample size, *SCZ* schizophrenia, *SCZA* schizoaffective disorder, *PANSS* Positive and Negative Syndrome scale, *t* (independent samples *t*-test), *x*^2^ (chi-square test), degrees of freedom (*df*), *NA* not applicable, *SD* standard deviation

^a^Years of education: calculated as the years of education after compulsory education (minimum compulsory education in the United Kingdom is 12 years)

^b^Socio-economic status: High = high, intermediate and lower grade professionals; medium = small employer, self-employed and lower technical occupations; low = sales, routine occupations, unemployed; student = student

### Sternberg Item-Recognition fMRI Paradigm

#### Performance

There were no significant group differences in performance accuracy on load 1 (t(64) = 1.44, *p* = 0.15) or 7 (t(64) = 1.95, *p* = 0.06) but there were group differences on loads 3 (t(64) = 2.32, *p* = 0.024), 5 (t(64) = 2.69, *p* = 0.01) and 9 (t(64) = 2.70, *p* = 0.01). These findings did not survive Bonferroni corrections. Relative to controls (M = 0.14 mm; SD = 0.07), patients (M = 0.21 mm; SD = 0.17) showed greater total frame-wise displacement (t(64) = −2.12, *p* = 0.04).

#### fMRI analyses

##### Data normality and equality of variance

All fMRI data were normally distributed. There were no differences in the variances for fMRI data between groups (see supplementary table 1).

##### Encoding

In a whole-brain analysis of encoding trials (35 controls, 31 patients), there was a significant main effect of group in the left angular gyrus, left superior parietal lobe; and a significant main effect of WM load in the lingual gyrus and the posterior cingulate gyrus (see Supplementary Table 2). These findings remained unchanged when restricting the analysis to male volunteers (26 controls, 26 patients) (see Supplementary Table 3).

In a whole-brain analysis of encoding trials (PET-fMRI subset), there was a significant main effect of group in the right middle temporal gyrus and frontal gyrus; and a significant main effect of WM load in the left superior parietal gyrus and the left middle frontal gyrus (see Supplementary Table 4). In ROI analyses of encoding trials, there was a significant main effect of group in the right hippocampus, left DLPFC and the left ACC; and a significant main effect of WM load in the left striatum, bilateral DLPFC and the bilateral ACC (see Table 2 for full results).

**Table 2:** Region of interest analysis of the effects of working memory encoding and working memory retrieval in all male healthy volunteers (*N* = 20) and male patients with first episode psychosis (*N* = 20) who had PET and fMRI when controlling for age, sex and frame-wise displacement.

Group	Contrast	ROI	H	MNI coordinates	F	Z	CS	p*
Healthy volunteers vs. patients	Encoding: main effect of group	Bilateral hippocampus	R	26 –8 –20	12.50	3.27	2	0.039
	Encoding: main effect of load	Bilateral hippocampus	NA	NA	NA	NA	NA	NA
	Encoding: group × load interaction	Bilateral hippocampus	NA	NA	NA	NA	NA	NA
Healthy volunteers vs. patients	Encoding: main effect of group	Bilateral striatum	NA	NA	NA	NA	NA	NA
	Encoding: main effect of load	Bilateral striatum	L	−20 6 2	9.77	4.37	118	0.003
	Encoding: main effect of load	Bilateral striatum	R	20 12 2	8.59	4.37	79	0.012
	Encoding: group × load interaction	Bilateral striatum	NA	NA	NA	NA	NA	NA
Healthy volunteers vs. patients	Encoding: main effect of group	Bilateral DLPFC	L	44 6 34	22.37	4.41	77	0.006
	Encoding: main effect of load	Bilateral DLPFC	L	−48 2 34	19.20	6.31	273	<0.001
	Encoding: main effect of load	Bilateral DLPFC	R	8 24 36	13.10	5.16	85	<0.001
	Encoding: main effect of load	Bilateral DLPFC	R	50 6 28	9.72	4.35	84	0.009
	Encoding: group × load interaction	Bilateral DLPFC	NA	NA	NA	NA	NA	NA
Healthy volunteers vs. patients	Encoding: main effect of group	Bilateral ACC	L	−10 36 20	16.52	3.78	21	0.017
	Encoding: main effect of load	Bilateral ACC	R	8 18 38	13.97	5.35	384	<0.001
	Encoding: group × load interaction	Bilateral ACC	NA	NA	NA	NA	NA	NA
Healthy volunteers vs. patients	Retrieval: main effect of group	Bilateral Hippocampus	R	26 –26 –12	25.14	4.67	79	<0.001
	Retrieval: main effect of group	Bilateral Hippocampus	L	−30 –30 –12	22.02	4.38	49	0.001
	Retrieval: main effect of load	Bilateral Hippocampus	NA	NA	NA	NA	NA	NA
	Retrieval: group × load interaction	Bilateral Hippocampus	NA	NA	NA	NA	NA	NA
Healthy volunteers vs. patients	Retrieval: main effect of group	Bilateral striatum	R	30 2 –8	34.32	5.43	201	<0.001
	Retrieval: main effect of group	Bilateral striatum	L	−30 –6 –8	25.94	4.75	35	<0.001
	Retrieval: main effect of group	Bilateral striatum	L	−14 12 14	21.94	4.37	57	0.002
	Retrieval: main effect of group	Bilateral striatum	R	16 6 16	21.21	4.29	80	0.003
	Retrieval: main effect of load	Bilateral striatum	NA	NA	NA	NA	NA	NA
	Retrieval: group × load interaction	Bilateral striatum	NA	NA	NA	NA	NA	NA
Healthy volunteers vs. patients	Retrieval: main effect of group	Bilateral DLPFC	R	2 42 26	22.40	4.41	176	0.005
	Retrieval: main effect of group	Bilateral DLPFC	R	46 2 32	19.20	4.09	28	0.018
	Retrieval: main effect of load	Bilateral DLPFC	NA	NA	NA	NA	NA	NA
	Retrieval: group × load interaction	Bilateral DLPFC	NA	NA	NA	NA	NA	NA
Healthy volunteers vs. patients	Retrieval: main effect of group	Bilateral ACC	R	10 0 42	24.54	4.62	154	0.001
	Retrieval: main effect of group	Bilateral ACC	R	10 42 12	20.77	4.25	167	0.002
	Retrieval: main effect of group	Bilateral ACC	L	−8 16 28	19.06	4.07	43	0.005
	Retrieval: main effect of load	Bilateral ACC	R	10 24 32	8.06	3.88	19	0.011
	Retrieval: main effect of group	Bilateral ACC	L	−4 22 38	7.11	3.58	5	0.031
	Retrieval: group × load interaction	Bilateral ACC	NA	NA	NA	NA	NA	NA

*H* hemisphere, *L* left, *R* right, *MNI* Montreal Neurological Institute, *CS* cluster size, p* *p* value surviving family-wise error (FWE) correction on the basis of peak-level extent

##### Retrieval

**Fig. 1:**
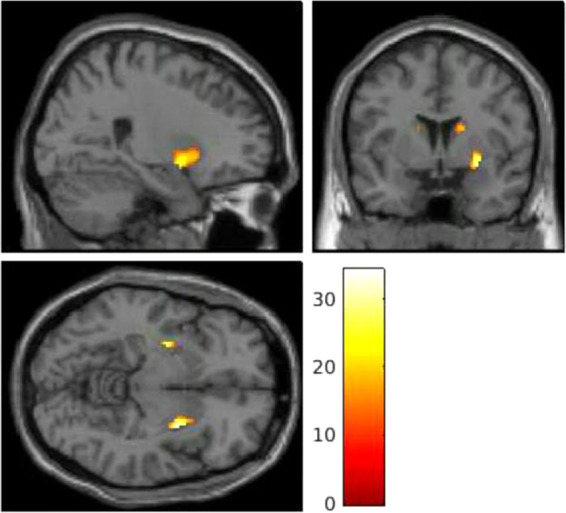
Statistical parametric maps showing that a main effect of group in the striatum during working memory in first episode psychosis patients relative to healthy volunteers ((MNI coordinates: *x* = 30, *y* = 2, *z* = −8); F =  34.32, *Z* = 5.43, cluster size = 201, *p* < 0.001). These findings survived family-wise error (FWE) correction on the basis of the peak-level extent (pFWE < 0.05). The colour bar shows the *t* statistic.

In a whole-brain analysis of retrieval trials (35 controls, 31 patients), there was a significant main effect of group in the bilateral hippocampus and the left posterior cingulate (see Supplementary Table 2). These findings remained unchanged when restricting the analysis to male volunteers (26 controls, 26 patients) (see Supplementary Table 3). In a whole-brain analysis of retrieval trials (PET-fMRI subset), there was a significant main effect of group in the left hippocampus, bilateral middle temporal gyrus, left superior temporal gyrus, bilateral putamen, right anterior cingulate gyrus and the left caudate (see Supplementary Table 4). In ROI analyses of retrieval trials, there was a significant main effect of group in the bilateral striatum (see Fig. 1), bilateral hippocampus, right DLPFC and bilateral ACC (see Table 2 for results).

### CB1R availability

Data were normally distributed. There was no difference in the variances between groups (see Supplementary Table 1). In a 2 (group) × 4 (region: DLPFC, ACC, hippocampus, striatum) repeated measures ANOVA, there was a significant effect of group (F(1,38) = 4.61, *p* = 0.03) and region (F(1,38) = 27.43, *p* < 0.001). However, the group × load interaction was not significant (F(1,38) = 0.47, *p* = 0.50).

### Association between CB1R availability and working memory

**Fig. 2:**
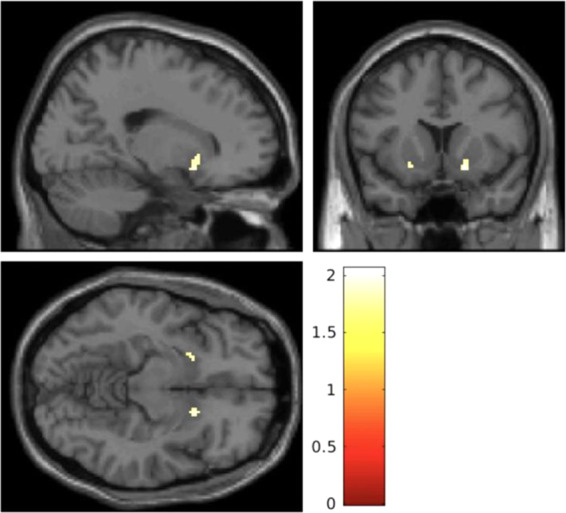
Statistical parametric maps showing that cannabinoid 1 receptor availability, as determined by the distribution volume of [^11^C] MePPEP, is significantly lower in the striatum in patients relative to controls. Results are show using a height threshold *p* < 0.05 for visualization purposes. The colour bar shows the *t* statistic.

**Fig. 3:**
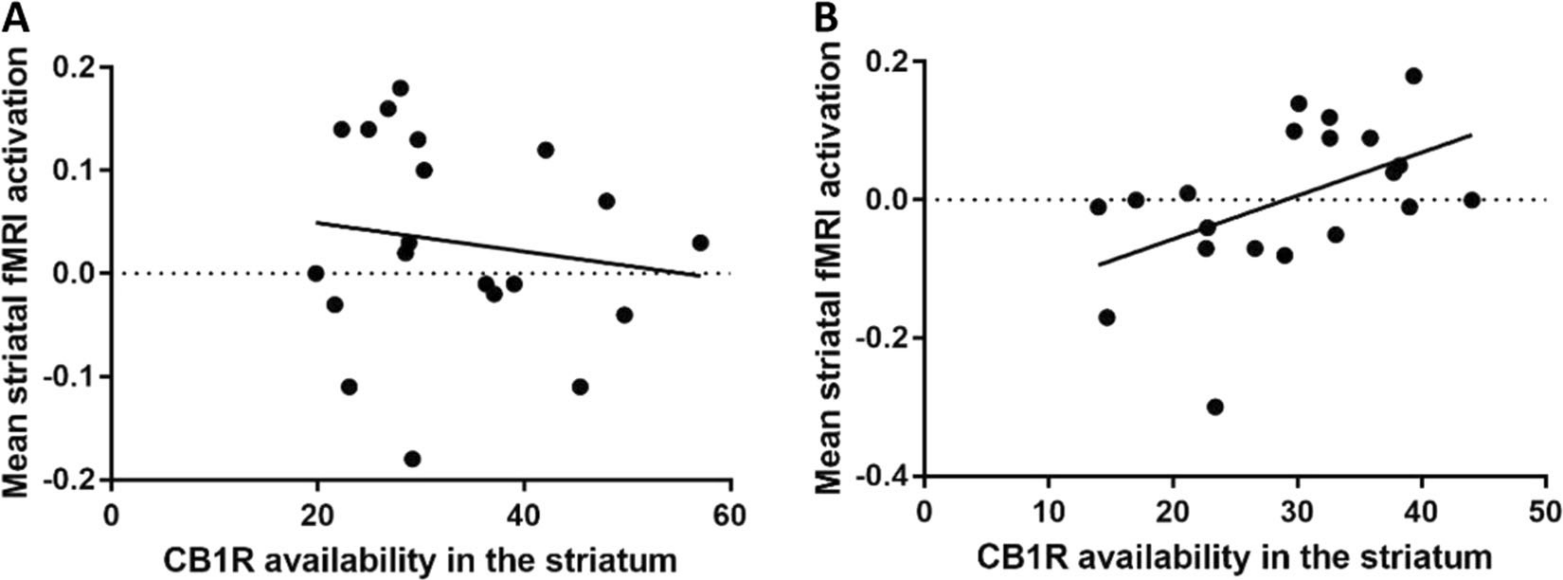
The associtation between striatal CB1R availability and the striatal neural correlates of working memory retrieval. **a** Association between the distribution volume of [^11^C]MePPEP in the striatum (ml/cm^3^) and mean load-dependent striatal BOLD signal during WM retrieval (beta values) during working memory in the striatum in healthy volunteers and **b** patients with first episode psychosis. A Fisher *r*-to-*z* transformation indicated that these relationships were significantly different between groups, *Z* = −2.20, *p* = 0.02 (two-tailed).

There were no significant associations between performance accuracy and CB1R availability (see supplementary materials). However, male patients showed a significant positive association between striatal CB1R availability (see Fig. 2) and mean linear load-dependent responses of striatal BOLD signal during WM retrieval (*R* = 0.50, *p* = 0.02; see Figs. 2–3) but male controls did not (*R* = −0.15, *p* = 0.53). However, neither of these findings survived Bonferroni corrections. The association in patients remained significant, when controlling for mean performance accuracy (*R* = 0.39, *p* = 0.04). However, this association fell short of statistical significance (*p* = 0.05) when restricting the analysis to volunteers who had PET and MRI scans <10 days apart (see supplementary materials). There were no other significant associations (see Supplementary Tables 5–6 for full results).

## Discussion

Our main finding was that male patients relative to male controls showed altered functional activation during WM encoding in the DLPFC, ACC and hippocampus and altered functional activation during WM retrieval in the DLPFC, ACC, hippocampus and striatum. Interestingly, the same male patients showed fewer cannabinoid 1 receptor levels in these brain regions relative to healthy volunteers. Moreover, male patients showed a positive association between striatal CB1R availability and load-dependent functional activation in the striatum during WM retrieval.

Consistent with previous literature [6], WM encoding was associated with greater task-related BOLD responses in the hippocampus and striatum. However, our finding that controls showed greater striatal and hippocampal activation during WM retrieval was not shown previously [6]. Instead, greater task-related BOLD responses during WM retrieval in the parietal, temporal and cingulate cortices have been reported in controls [6]. Since we used peak-level thresholding, this discrepant finding may be explained by the use of cluster-extent thresholding in this previous study, shown to have poor spatial specificity and increase false positive rates [44].

Our finding that patients showed greater activation during WM retrieval in the left hippocampus and the left caudate in drug-free patients is consistent with findings in anti-psychotic-treated, chronic male patients [12]. However, our finding extends this work by showing that drug-free/naïve FEP patients show altered activation during WM retrieval. Although our finding that patients show greater activation in the middle frontal gyrus during WM encoding is at odds with literature reporting decreased activation in this region during WM encoding [12], this previous study showed group differences in performance accuracy which may indicate poor task engagement in patients [12].

Our finding that patients showed lower CB1R availability in regions implicated in memory [6] is consistent with previous work using arterial blood sampling [15, 22], but is at odds with a study that failed to use arterial blood sampling [45], necessary for the reliable estimation of the radiotracer kinetics [43]. Our finding that patients show lower CB1R levels, in the context of functional alterations during WM, is consistent with literature demonstrating that CB1R-deficient mice exhibit WM deficits [13, 17]. Moreover, our finding that CB1R levels were not associated with behavioural measures of WM is also consistent with literature showing that peripheral CB1R mRNA levels were not associated with WM [14].

A recent meta-analysis indicated that central levels of CB1R agonist, anandamide, are elevated in patients with psychotic illnesses [46]. Moreover, the acute administration of partial CB1R agonist, delta-9-tetrahydrocannabinol, consistently induced WM deficits in healthy volunteers in a systematic review of 35 human studies [17]. Previous work has also shown that delta-9-tetrahydrocannabinol induces greater WM deficits in schizophrenia relative to healthy individuals [47]. While it is unclear if higher levels of endogenous CB1R agonists precipitate CB1R down-regualtion in psychosis, the chronic exposure to CB1R agonists, delta-9-tetrahydrocannabinol, down-regulates CB1R levels in humans. Moreover, CB1R knockout mice, who are deficient in CB1R on GABAergic interneurons, show WM deficits [13].

### Strengths and limitations

A strength of the study was that patients were anti-psychotic naïve/free and satisfied criteria for schizophrenia or schizoaffective disorder, shown to have good diagnostic stability [48, 49]. A limitation of our study, inherent to all cross-sectional designs, is that we are unable to determine if CB1R alterations are causally implicated in cognitive impairments. Future longitudinal studies using pharmacological interventions, that modulate CB1R availability, are needed to determine whether CB1R agonist-mediated reductions in CB1R availability [50] induce WM impairments in humans. A limitation of the study was that CB1R availability was only investigated in male volunteers. Given sex differences in CB1R availability [26] and sex differences in the behavioural and functional effects of cannabinoids on neuronal excitability and synaptic plasticity [51], we specifically investigated the association between CB1R and WM in males to reduce the effect of sex as a source of variability. However, future studies are needed to determine if our findings generalize to female patients.

PET and MRI scans were conducted as closely together as possible. However, due to limited scanner availability, these scans were approximately 1 month apart. Since test−retest data indicates that CB1R availability, as determined by the VT of [^11^C]MePPEP, remains stable between 1 and 309 days [32], the delay between the two scans is unlikely to impact the variability of the VT. When restricting the analysis to volunteers who had PET and MRI scans less than 10 days apart, we observed a trend-level association between striatal CB1R availability and mean linear load-dependent BOLD responses in the striatum during memory retrieval. This association is likely to have fallen short of statistical significance (*p* = 0.05) since the sample was statistically underpowered, in accordance with our power calculation.

Since the endocannabinoid system dynamically changes in response to cannabis use [50], we ensured that all subjects had negative urine drug screens prior to both scans and that subjects with current substance or a history of substance abuse/dependence were excluded. However, future studies should use simultaneous PET-fMRI scanners to improve the integration of multimodal imaging data.

While some subjects had previously used cannabis, 1 month of abstinence normalizes CB1R levels [52] and there were no associations between CB1R levels and prior cannabis use [15]. Similarly, although tobacco use may influence CB1R levels [53], there were no group differences in tobacco use or associations between CB1R levels and tobacco use [15].

Although we were able to disentangle the effects of WM encoding and WM retrieval, WM maintenance was not modelled. Future studies could address this by imposing a longer delay between encoding and retrieval blocks. Although performance was not included in the model, the association between striatal CB1R availability and striatal fMRI BOLD responses remained unchanged when controlling for performance. However, this finding did not survive Bonferroni corrections for multiple comparisons. Moreover, while patients showed greater frame-wise displacement relative to controls, we controlled for frame-wise displacement in all fMRI analyses and thus, this is unlikely to be a significant confound.

Given findings indicating that behavioural and functional measures of WM are associated with functional polymorphisms in the cannabinoid 1 receptor (*CNR1*) gene [54, 55], a limitation was that we were unable to investigate functional polymorphisms in the *CNR1* gene and how they may be linked to CB1R and WM in schizophrenia.

### Implications for understanding the neurobiology of working memory deficits

Our finding that patients show greater activation during both WM encoding and WM retrieval, in the context of no significant differences in performance, suggests that patients may utilize greater levels of neural activity to achieve levels of performance comparable to controls.

Our findings show that WM encoding and WM retrieval processes are both altered in the early stages of schizophrenia without the confounds of substance use [56] and anti-psychotic medication [12]. Moreover, our finding that striatal CB1R availability is associated with altered striatal fMRI activation during WM retrieval in drug-naïve/free FEP patients extends preclinical literature demonstrating that CB1R agonists administered to the striatum impair memory [39, 40]. Since this association was exclusively shown in patients, but not controls, striatal CB1R dysfunction may precipitate an adaptation in the normal mechanisms underlying WM retrieval in the early stages of psychosis.

Our finding that striatal CB1R availability was associated with striatal activation during WM retrieval may be due to the unique topographical organization of striatal CB1R. CB1R are densely distributed on GABAergic interneurons in the striatum, where they inhibit GABA release [57, 58], a mechanism known as depolarization-induced suppression of inhibition [59]. By contrast, CB1R are predominately localized on glutamate neurons in the hippocampus [60] and pyramidal neurons in the cortex [61], where they inhibit glutamate release, a mechanism known as depolarization-induced suppression of excitation [59]. The regional specificity of our findings may therefore indicate that WM impairments in schizophrenia are linked to alterations in the disinhibition of synaptic transmission, arising from striatal CB1R dysfunction on GABAergic interneurons. This adds to other neurochemical evidence implicating the striatum in the pathophysiology of schizophrenia [62–66].

Since CB1R regulate neurotransmitter release by inhibiting N-, P- and Q-type calcium channel openings and by activating inwardly rectifying potassium channels [67], fewer CB1R may disrupt the balance of excitatory and inhibitory synaptic transmission underlying long-term potentiation [24, 68]. These findings identify the CB1R as potential target for the treatment of WM impairments in FEP.

## Conclusions

Relative to controls, drug-naïve/free FEP patients exhibit functional alterations during WM encoding and WM retrieval. In contrast to controls, patients showed a positive association between striatal CB1R availability and mean load-dependent striatal functional activation during WM retrieval. These findings identify altered striatal CB1R availability and striatal neural correlates of WM retrieval in the pathophysiology of WM impairments in FEP.

## Supplementary information

Supplementary Material

## Data Availability

All codes are available upon request.
