# Supplementary material for: The neural and molecular basis of working memory function in psychosis: a multimodal PET-fMRI study
Source: Mol Psychiatry. 2019 Dec 4;26(8):4464–74. doi: 10.1038/s41380-019-0619-6 (PMC8550949; doi:10.1038/s41380-019-0619-6)
Supplement: Supplementary file 1 — Supplementary Material [file 41380_2019_619_MOESM1_ESM.docx]

**Supplementary materials**

***Sample size and power calculation***

We determined the minimum sample size necessary to test our primary hypothesis that CB1R would be associated with the neural correlates of working memory. Since no studies have investigated this in animals or humans, we based our power calculation on a previous study investigating the association between CB1R, as measured by [^11^C]MePPEP, and a cognitive emotional processing paradigm (Bhattacharyya et al. 2017). This previous study found a relationship between these measures, with an R^2^=0.47 and a sample size of 14. A power calculation in G*power version 3.1 (<https://download.cnet.com/G-Power/>) indicated that a sample size of 20 subjects per group would have greater than 80% power to detect a relationship of R^2^=0.35 or greater using biserial correlation and for p<0.05 (two-tailed).

***Neuroimaging acquisition parameters***

***Structural magnetic resonance imaging***

High-resolution 3D SPGR T1 images were acquired (in-plane matrix size of 256 × 256, FOV = 26.0 mm) using a whole-brain, interleaved bottom-up acquisition using a sagittal orientation and an 8-channel head coil (TR = 7.34 ms, TE = 3.036 ms, inversion time = 4 seconds, flip angle = 11°, slice thickness = 1.2 mm, slice gap = 1.2 mm). The sequence duration was 14 minutes and 6 seconds.

***Functional magnetic resonance imaging acquisition***

287 EPI 2D Gradient Echo volumes were collected during the Sternberg working memory fMRI paradigm (in-plane matrix size of 64 × 64 and a field of view [FOV] of 21.1 mm), using a whole-brain, sequential top-down acquisition for each functional time point using an oblique orientation and an 8-channel head coil (TR = 2 s, TE =30 ms, flip angle = 75°, 39 slices per volume, slice thickness = 3 mm, between-slice gap = 3.3mm). The sequence duration was 11 minutes.

***Functional magnetic resonance imaging: Sternberg working memory paradigm***Encoding trials consisted of the presentation of a letter set (6600 ms) followed by the presentation of a fixation cross (600 ms). Encoding trials varied in their working memory load demands, where the letter sets consisted of either 1, 3, 5, 7 or 9 consecutive consonants. Retrieval trials consisted of the simultaneous presentation of a 1) individual letter and 2) two text boxes (“yes” and “no”) (1500ms), followed by the presentation of a fixation cross (900ms). The text boxes (“yes” and “no”) could be selected by pressing on a two-button box using left and right buttons, respectively. The rest trials (N=4) consisted of the presentation of a fixation cross (10000ms). Each encoding trial was followed by eight retrieval trials. The task consisted of 20 encoding trials (7200ms each), 160 retrieval trials (2400ms each) and 4 rest trials (10000ms each).

***Positron emission tomography***A continuous 90-minute PET scan was conducted using a Hi-Rez Biograph 6 CT44931 scanner in three-dimensional mode, following a bolus injection of 314 ± 34.4 MBq of [^11^C]MePPEP using discrete and continuous arterial blood sampling (see supplementary materials for details). CT scans were acquired prior to each PET scan for attenuation correction. Images were reconstructed with filtered back projection including corrections for attenuation, random and scatter. The mean [SD] duration between MRI and PET scans was 35.72 [45.37] days. The quantification of the PET images and the estimation of the VT values were completed using in-house scripts using Matlab 8.5 (The Mathworks 2010) that are available upon request.


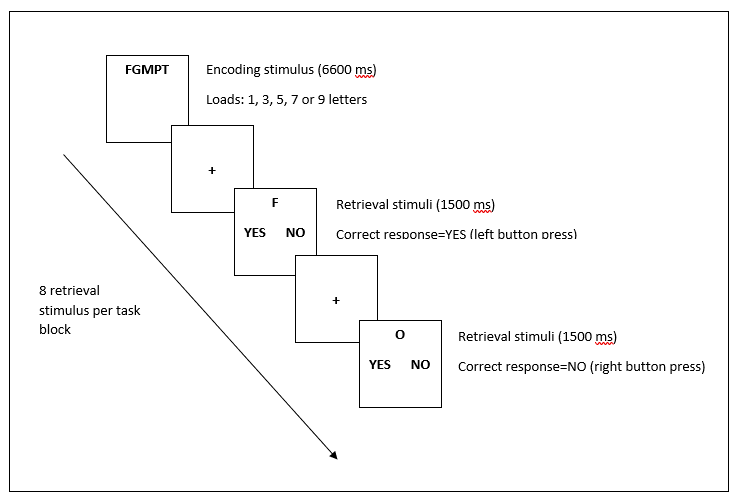


**Supplementary figure 1. Schematic of the Sternberg Item-Recognition Paradigm measuring working memory. The encoding condition involves the presentation of letter sets (6600 ms), varying in their working memory load demands (1, 3, 5, 7 or 9 letters). The retrieval condition consisted of the presentation of retrieval stimuli (8 letters per task block). Volunteers are instructed to indicate whether individual letters were presented in the previous letter set (Target=YES, Non-target=NO).**

***MRI image analysis***

Structural T1-weighted structural scans were segmented, warped to a template created using the DARTEL algorithm which improves the accuracy of inter-subject registration and realignment, normalized into MNI space and smoothed using an 8mm Gaussian kernel. To identify if there were volumetric differences between patients and controls, global tissues volumes were compared using an independent samples t-test was conducted in SPM12 including age and total intracranial volume as covariates.

***fMRI image analysis***

The pre-processing pipeline consisted of the manual reorienting of the structural and functional images so that the anterior commissure lies on the origin (coordinate [0 0 0]), slice timing, head motion correction, co-registration of the functional image to the structural file, segmentation, normalization to MNI space and smoothing using 8-m Gaussian kernel to minimize noise and residual differences in gyral anatomy.

***PET image analysis***

Data pre-processing was performed using a combination of Statistical Parametric Mapping 8 (<http://www.fil.ion.ucl.ac.uk/spm>) and FSL (<http://www.fsl.fmrib.ox.ac.uk/fsl>) functions, as implemented in MIAKAT ([miakat.org)](http://www.imanova.co.uk)). Motion correction was applied to non-attenuation corrected images (Montgomery et al. 2006). Non-attenuated corrected frames were realigned to a single “reference” frame (corresponding to that with the highest number of counts) by employing a mutual information algorithm. The transformation parameters were then applied to the corresponding attenuated-corrected dynamic images, creating a movement-corrected dynamic image which was used for the analysis. Realigned frames were then summated to create an individual motion-corrected reference map for the brain tissue segmentation. T1-weighted structural images were co-registered to the PET image using rigid body transformation. Normalization parameters were obtained by warping the co-registered structural MRI to MNI space (International Consortium for Brain Mapping ICBM/MNI). The inverse of these parameters was used to fit a neuroanatomical atlas to each individual PET scan using the Hammersmith atlas (Hammers et. al 2003). An extended version of Hill model (Edison, P., Brooks, D.J., Turkheimer, F.E., Archer, H.A., Hinz 2009) was selected as the one leading to the best description of arterial input function data, in agreement with previous literature (Tonietto, M., Veronese, M., Rizzo, G., Zanotti-Fregonara, P., Lohith, T. G., Fujita, M., ... & Bertoldo 2015; Tonietto et al. 2018)(Tonietto, M., Veronese, M., Rizzo, G., Zanotti-Fregonara, P., Lohith, T. G., Fujita, M., ... & Bertoldo 2015). Similarly, whole blood time-activity curves (TACs) were fitted using a multi-exponential function as derived by Feng’s model (Feng, Huang, and Wang 1993). In addition, for each scan, a time delay was fitted and applied to the input functions (both parent and whole blood TACs) to account for any temporal delay between blood sample measurement and target the tissue data.

**Supplementary table 1. Data variance and equality of variance results for functional magnetic resonance imaging and positron emission tomography data**

| **Variable** | **Healthy volunteers** | **Patients** | **F** | **Df** | **p** |
| --- | --- | --- | --- | --- | --- |
| CB1R availability in the striatum | SD=10.52 | SD=8.71 | 1.03 | 38 | 0.32 |
| CB1R availability in the anterior cingulate | SD=10.71 | SD=9.48 | 0.25 | 38 | 0.62 |
| CB1R availability in the dorsolateral prefrontal cortex | SD=3.34 | SD=2.88 | 0.25 | 38 | 0.62 |
| CB1R availability in the hippocampus | SD=10.91 | SD=11.14 | 0.01 | 38 | 0.93 |
| fMRI mean load-dependent whole-brain activation during encoding | SD=2.43 | SD=2.76 | 0.01 | 64 | 0.94 |
| fMRI mean load-dependent whole-brain activation during retrieval | SD=1.02 | SD=1.00 | 0.11 | 64 | 0.74 |

**SD=standard deviation; F=Lavene’s Test of Equality of Variance**

**Supplementary table 2. Whole-brain analysis results of the effects of memory encoding and retrieval in all healthy volunteers (N=35) and patients with first episode psychosis (N=31) when controlling for age, sex and frame-wise displacement**

| **Contrast** | **Cluster** | **H** | **MNI coordinates** | **F** | **Z** | **CS** | **P*** |
| --- | --- | --- | --- | --- | --- | --- | --- |
| Encoding: main effect of group | Angular gyrus | L | -34 -60 26 | 26.21 | 4.85 | 130 | 0.003 |
|  | Middle occipital gyrus | R | 36 -60 14 | 25.09 | 4.75 | 54 | 0.005 |
|  | Superior parietal lobe | L | -26 -70 36 | 21.22 | 4.36 | 46 | 0.02 |
|  | Occipital fusiform gyrus | R | 28 -76 -26 | 20.87 | 4.32 | 163 | 0.03 |
| Encoding: Main effect of load | Lingual gyrus | L | -14 -86 -6 | 29.60 | Inf | 9936 | <0.001 |
|  | Posterior cingulate gyrus | L | -12 -50 30 | 10.03 | 4.54 | 400 | 0.01 |
| Encoding: group x load interaction | No suprathreshold clusters |  |  |  |  |  |  |
| Retrieval: main effect of group | Hippocampus | L | -24 -6 -12 | 29.81 | 5.18 | 74 | 0.001 |
|  | Hippocampus | R | 24 -4 -12 | 28.08 | 5.02 | 78 | 0.001 |
|  | Posterior cingulate gyrus | L | -20 -42 30 | 27.63 | 4.98 | 437 | 0.002 |
|  | Parietal operculum | L | -32 -36 16 | 22.37 | 4.48 | 68 | 0.01 |
| Retrieval: main effect load | No suprathreshold clusters |  |  |  |  |  |  |
| Retrieval: group x load interaction | No suprathreshold clusters |  |  |  |  |  |  |

Abbreviations: H=hemisphere; L=left; R=right; MNI=Montreal Neurological Institute; CS=cluster size; p*=p value surviving family-wise error (FWE) correction on the basis of peak-level extent.

**Supplementary table 3. Whole-brain analysis results of the effects of memory encoding and retrieval in all male healthy volunteers (N=26) and all male patients with first episode psychosis (N=26) when controlling for age and frame-wise displacement**

| **Contrast** | **Cluster** | **H** | **MNI coordinates** | **F** | **Z** | **CS** | **P*** |
| --- | --- | --- | --- | --- | --- | --- | --- |
| Encoding: main effect of group | Superior parietal lobe | L | -26 -72 36 | 26.41 | 4.84 | 57 | 0.003 |
|  | Angular gyrus | L | -30 -56 22 | 21.20 | 4.33 | 49 | 0.027 |
|  | Lingual gyrus | R | 32 -48 2 | 21.17 | 4.33 | 69 | 0.027 |
| Encoding: Main effect of load | Lingual gyrus | L | -14 -86 -6 | 21.39 | 6.81 | 7386 | <0.001 |
|  | Precuneus, posterior cingulate gyrus | L | -12 -50 30 | 10.01 | 4.49 | 339 | 0.017 |
| Encoding: group x load interaction | No suprathreshold clusters |  |  |  |  |  |  |
| Retrieval: main effect of group | Hippocampus, Pallidum | R | 24 -4 -12 | 32.3- | 5.34 | 109 | <0.001 |
|  | Precuneus, posterior cingulate | L | -4 -62 18 | 26.58 | 4.85 | 2040 | 0.003 |
|  | Amygdala | L | -24 -6 -12 | 25.57 | 4.76 | 71 | 0.005 |
| Retrieval: main effect load | No suprathreshold clusters |  |  |  |  |  |  |
| Retrieval: group x load interaction | No suprathreshold clusters |  |  |  |  |  |  |

Abbreviations: H=hemisphere; L=left; R=right; MNI=Montreal Neurological Institute; CS=cluster size; p*=p value surviving family-wise error (FWE) correction on the basis of peak-level extent.

**Supplementary table 4. Whole-brain analysis results of the effects of memory encoding and retrieval in male healthy volunteers (N=20) and male patients with first episode psychosis (N=20) who had PET and fMRI when controlling for sex and frame-wise displacement**

| **Contrast** | **Cluster** | **H** | **MNI coordinates** | **F** | **Z** | **CS** | **P*** |
| --- | --- | --- | --- | --- | --- | --- | --- |
| Encoding: main effect of group | Middle temporal gyrus | R | 52 -38 -4 | 27.68 | 4.90 | 128 | 0.007 |
|  | Triangular part of inferior frontal gyrus | R | 44 34 0 | 27.19 | 4.86 | 196 | 0.008 |
|  | Middle frontal gyrus | R | 40 4 34 | 24.04 | 4.57 | 137 | 0.027 |
| Encoding: main effect of load | Superior parietal lobe | L | -22 -62 44 | 19.89 | 6.42 | 4349 | <0.001 |
|  | Middle frontal gyrus | L | -48 2 34 | 19.20 | 6.31 | 2927 | <0.001 |
|  | Middle occipital gyrus | R | 30 -82 10 | 18.36 | 6.17 | 3746 | <0.001 |
|  | Middle frontal gyrus | R | 26 0 50 | 11.76 | 4.86 | 208 | 0.009 |
|  | Anterior insula | R | 32 22 2 | 10.66 | 4.60 | 519 | 0.02 |
|  | Precentral gyrus | R | 52 8 24 | 10.61 | 4.58 | 297 | 0.03 |
| Encoding: group x load interaction | No suprathreshold clusters |  |  |  |  |  |  |
| Retrieval: main effect of group | Hippocampus | L | -18 -40 -12 | 40.24 | 5.85 | 4888 | <0.001 |
|  | Middle temporal gyrus | R | 62 -32 -2 | 39.87 | 5.82 | 1067 | <0.001 |
|  | Superior temporal gyrus | L | -52 -8 -6 | 39.76 | 5.81 | 850 | <0.001 |
|  | Putamen | R | 30 2 -10 | 34.54 | 5.44 | 1201 | <0.001 |
|  | Precentral gyrus | L | -32 -26 48 | 28.94 | 5.00 | 419 | 0.003 |
|  | Middle temporal gyrus | L | -62 -40 -6 | 28.66 | 4.98 | 252 | 0.003 |
|  | Putamen | L | -30 -6 -8 | 25.94 | 4.75 | 139 | 0.01 |
|  | Anterior cingulate gyrus | R | 12 42 12 | 22.88 | 4.46 | 537 | 0.03 |
|  | Caudate | L | -14 12 14 | 21.94 | 4.37 | 61 | 0.04 |
| Retrieval: main effect of load | No suprathreshold clusters |  |  |  |  |  |  |
| Retrieval: group x load interaction | No suprathreshold clusters |  |  |  |  |  |  |

Abbreviations: H=hemisphere; L=left; R=right; MNI=Montreal Neurological Institute; CS=cluster size; p*=p value surviving family-wise error (FWE) correction on the basis of peak-level extent.

***Association between CB1R availability and working memory performance***Male controls showed no association between performance accuracy and CB1R availability in the DLPFC (R=0.23, p=0.34), ACC (R=0.34, p=0.15), striatum (R=-0.03, p=0.91) or hippocampus (R=-0.14, p=0.55). Similarly, male patients also showed no association between performance accuracy and mean CB1R availability in the DLPFC (R=0.43, p=0.06), ACC (R=0.35, p=0.13), striatum (R=0.35, p=0.13) or hippocampus (R=0.30, p=0.20).

**Supplementary table 5. Association between cannabinoid 1 receptor availability and the neural correlates of working memory in healthy volunteers**

| **Condition** | **PET region** | **fMRI region** | **R** | **p** |
| --- | --- | --- | --- | --- |
| Encoding | Striatum | Striatum | -0.29 | 0.22 |
| Retrieval | Striatum | Striatum | -0.15 | 0.53 |
| Encoding | Hippocampus | Hippocampus | -0.11 | 0.65 |
| Retrieval | Hippocampus | Hippocampus | 0.17 | 0.48 |
| Encoding | DLPFC | DLPFC | 0.19 | 0.43 |
| Retrieval | DLPFC | DLPFC | -0.03 | 0.91 |
| Encoding | ACC | ACC | -0.20 | 0.48 |
| Retrieval | ACC | ACC | -.10 | -0.10 |

**Supplementary table 6. Association between cannabinoids 1 receptor availability and the neural correlates of working memory in patients**

| **Condition** | **PET region** | **fMRI region** | **R** | **p** |
| --- | --- | --- | --- | --- |
| Encoding | Striatum | Striatum | 0.10 |  |
| Retrieval | Striatum | Striatum | 0.48 | 0.03* |
| Encoding | Hippocampus | Hippocampus | 0.08 | 0.73 |
| Retrieval | Hippocampus | Hippocampus | 0.23 | 0.32 |
| Encoding | DLPFC | DLPFC | 0.17 | 0.47 |
| Retrieval | DLPFC | DLPFC | -0.001 | 0.99 |
| Encoding | ACC | ACC | 0.10 | 0.67 |
| Retrieval | ACC | ACC | 0.35 | 0.13 |

**Association between performance accuracy and the neural correlates of working memory**

**Healthy volunteers**Healthy volunteers showed no significant associations between mean performance accuracy and mean BOLD signal during working memory encoding in the hippocampus (R=0.20, p=0.40), striatum (R=0.24, 0.30), DLPFC (R=-0.08, p=0.73) or ACC (R=-0.20, p=0.46). Similarly, healthy volunteers showed no significant associations between mean performance accuracy and mean BOLD signal during working memory retrieval in the hippocampus (R=-0.28, p=0.23), striatum (R=-0.40, p=0.08), DLPFC (R=-0.08, p=0.73) or the ACC (R=-0.20, p=0.46).

**Patients**
Patients showed a significant association between mean performance accuracy and mean BOLD signal during working memory encoding in the striatum (R=0.50, p=0.02) but not the hippocampus (R=-0.30, p=0.23), DLPFC (R=0.25, p=0.28) or ACC (R=0.10, p=0.67). There were no significant associations between mean performance accuracy and mean BOLD signal during working memory retrieval in the striatum (R=0.08), hippocampus (R=0.33, p=0.16), DLPFC (R=0.24, p=0.30) or ACC (R=0.37, p=0.11).

***Association between CB1R availability and neural correlates of working memory***Our findings in healthy volunteers remained unchanged when restricting the analysis to volunteers who had the PET and MRI scans less than 10 days apart (N=7). Similarly, patients who had PET scans less than 10 days apart (N=10) showed a trend-level positive association between CB1R availability in the striatum and mean linear load-dependent BOLD responses during WM retrieval in the striatum (R=0.51, p=0.05). Moreover, CB1R availability in the DLPFC was trend-level associated with mean linear load-dependent BOLD responses in the DLPFC during WM retrieval (R=0.51, p=0.06).

***References***

Bhattacharyya, Sagnik, Alice Egerton, Euitae Kim, Lula Rosso, Daniela Riano Barros, Alexander Hammers, Michael Brammer, Federico E. Turkheimer, Oliver D. Howes, and Philip McGuire. 2017. “Acute Induction of Anxiety in Humans by Delta-9-Tetrahydrocannabinol Related to Amygdalar Cannabinoid-1 (CB1) Receptors.” *Scientific Reports* 7 (1). https://doi.org/10.1038/s41598-017-14203-4.

Edison, P., Brooks, D.J., Turkheimer, F.E., Archer, H.A., Hinz, R. 2009. “Strategies for the Generation of Parametric Images of [11C]PIB with Plasma Input Functions Considering Discriminations and Reproducibility.” *Neuroimage* 48: 329–38.

Feng, D, S C Huang, and X Wang. 1993. “Models for Computer Simulation Studies of Input Functions for Tracer Kinetic Modeling with Positron Emission Tomography.” *International Journal of Bio-Medical Computing* 32 (2): 95–110. http://www.ncbi.nlm.nih.gov/pubmed/8449593.

Montgomery, Andrew J, Kris Thielemans, Mitul A Mehta, Federico Turkheimer, Sanida Mustafovic, and Paul M Grasby. 2006. “Correction of Head Movement on PET Studies: Comparison of Methods.” *J Nucl Med* 47: 1936–44. https://tauruspet.med.yale.edu/staff/edm42/papers/journal-club/Montgomery_JNM_2006.pdf.

The Mathworks, Inc. 2010. “MATLAB.” Natick, Masaechusetts: Mathworks, Inc.

Tonietto, M., Veronese, M., Rizzo, G., Zanotti-Fregonara, P., Lohith, T. G., Fujita, M., ... & Bertoldo, A. 2015. “Improved Models for Plasma Radiometabolite Correction and Their Impact on Kinetic Quantification in PET Studies.” *Journal of Cerebral Blood Flow and Metabolism* 35 (9): 1462–69.

Tonietto, Matteo, Gaia Rizzo, Mattia Veronese, Faith Borgan, Peter Bloomfield, Oliver Howes, and Alessandra Bertoldo. 2018. “A Unified Framework for Plasma Data Modelling in Dynamic Positron Emission Tomography Studies.” *IEEE Transactions on Biomedical Engineering* 9294 (c): 1–1. https://doi.org/10.1109/TBME.2018.2874308.
